# Development and validation of a machine learning method to predict intraoperative red blood cell transfusions in cardiothoracic surgery

**DOI:** 10.1038/s41598-022-05445-y

**Published:** 2022-01-25

**Authors:** Zheng Wang, Shandian Zhe, Joshua Zimmerman, Candice Morrisey, Joseph E. Tonna, Vikas Sharma, Ryan A. Metcalf

**Affiliations:** 1 School of Computing, University of Utah, Salt Lake City, UT USA; 2 Department of Anesthesiology, University of Utah, Salt Lake City, UT USA; 3 Division of Cardiothoracic Surgery, Department of Surgery, University of Utah, Salt Lake City, UT USA; 4 Department of Pathology, University of Utah, Salt Lake City, UT USA; 5 ARUP Laboratories, Salt Lake City, UT USA

**Keywords:** Health care, Risk factors

## Abstract

Accurately predicting red blood cell (RBC) transfusion requirements in cardiothoracic (CT) surgery could improve blood inventory management and be used as a surrogate marker for assessing hemorrhage risk preoperatively. We developed a machine learning (ML) method to predict intraoperative RBC transfusions in CT surgery. A detailed database containing time-stamped clinical variables for all CT surgeries from 5/2014–6/2019 at a single center (n = 2410) was used for model development. After random forest feature selection, surviving features were inputs for ML algorithms using five-fold cross-validation. The dataset was updated with 437 additional cases from 8/2019–8/2020 for validation. We developed and validated a hybrid ML method given the skewed nature of the dataset. Our Gaussian Process (GP) regression ML algorithm accurately predicted RBC transfusion amounts of 0 and 1–3 units (root mean square error, RMSE 0.117 and 1.705, respectively) and our GP classification ML algorithm accurately predicted 4 + RBC units transfused (area under the curve, AUC = 0.826). The final prediction is the regression result if classification predicted < 4 units transfused, or the classification result if 4 + units were predicted. We developed and validated an ML method to accurately predict intraoperative RBC transfusions in CT surgery using local data.

## Introduction

Cardiothoracic (CT) surgeries are commonly performed and account for a significant proportion of red blood cell (RBC) transfusions that occur in the United States each year^1^. These cases are often complex with a substantial risk of significant hemorrhage and the most common setting of massive transfusion is CT surgery^2^. Multiple patient blood management (PBM) modalities exist to minimize hemorrhage and avoid unnecessary transfusions^3^. Consequently, using data to predict the risk of RBC transfusion in CT surgery patients has been of interest over the past two decades, which is evidenced by multiple previously published prediction models^4–8^.

Maximum surgical blood order schedules (MSBOSs) are a common strategy used by PBM programs to prevent excessive preoperative blood ordering. MSBOSs reduce unnecessary crossmatches, pre-transfusion testing, and costs, while possibly increasing the number of emergency release transfusions by a small amount^9^. However, MSBOSs are not designed to accurately predict RBC utilization for a given case. A natural evolution of the MSBOS could be more personalized predictions to further improve stewardship of pre-transfusion testing and the blood supply as well as the identification of cases at risk of significant hemorrhage and high blood utilization. Preoperative identification of these high-risk cases can improve preparation, encourage the use of PBM modalities, and potentially improve patient outcomes.

The performance of previously developed CT surgery RBC transfusion predictive models was typically reported using area under the curve (AUC) values^4–8^. While results suggested reasonable performance, there were limitations to each model. For example, some models used only binary classification to predict either zero units transfused or any transfusion amount greater than zero units^4,5,8^. Two of these studies predicted any perioperative transfusion rather than intraoperative transfusions, while another predicted any transfusion up to one day after the surgery. Two other studies attempted to predict larger transfusion amounts (e.g. > 4 or 5 RBCs transfused), but one predicted transfusion risk over the entire hospital stay and the other used intraoperative variables^6,7^. The most important predictor in this latter study was cardiopulmonary bypass time, which is not known preoperatively^7^. Therefore, these models provide limited information about preoperative blood ordering and intraoperative hemorrhage risk. Our aim was to use only preoperative variables to predict intraoperative blood use, thus informing more accurate preoperative blood ordering and providing a useful surrogate for risk and magnitude of intraoperative hemorrhage.

There are three major problems to consider when analyzing and predicting RBC use in CT surgery: (1) clinical data associated with each surgical case for each patient can be highly varied and voluminous, resulting in a long list of thousands of features; (2) the relationship between surgical data and the required amount of RBC components is expected to be complex and, therefore, a complex algorithm may be needed; (3) RBC utilization for different patients is highly varied, with the vast majority of patients using less than or equal to 3 units and a relatively small percentage using 4 + units, which can lead to highly biased predictions.

The development of machine learning (ML) algorithms for prediction in healthcare has garnered substantial interest^10^. With access to large, detailed, validated databases, an outcome of interest (e.g. mortality) can be predicted with excellent performance^11–16^. Unlike traditional statistical approaches, ML algorithms do not focus on a priori assumptions but rather learn from the dataset to achieve the most accurate output result. Further, ML algorithms vary in their complexity and transparency. A decision tree algorithm is a relatively simple ML approach that results in an intuitive and interpretable model, whereas neural networks may be highly complex and have less transparency (i.e. “black box”). The purpose of this study was to develop and validate a novel ML method to predict intraoperative allogeneic RBC transfusions in CT surgery and compare its performance to a spectrum of other well-known, commonly used ML algorithms.

## Methods

Records of all inpatient visits from May 2014 to July 2019 that involved a cardiothoracic (CT) surgery operating room procedure were obtained from and validated by the University of Utah Health’s Enterprise Data Warehouse (EDW). Detailed data were obtained on patient demographics, hospital visit characteristics, surgery cases, and all associated billing codes. Additionally, all laboratory values, vital signs, medications, and intraoperative blood transfusions along with associated dates and times were included (information about data tables—also known as business objects—is included in Supplementary Table S1). Of note, our protocol for blood transfusion in response to hemorrhage during CT surgery involves the interpretation of viscoelastic testing methods. Our local approach mirrors previously published approaches^17^. Our initial dataset’s CT surgery cases and associated features (variables) were used as predictive inputs into the models. Approval to perform this retrospective, observational study along with authorization of waived consent was obtained from the University of Utah School of Medicine Institutional Review Board (IRB). Methods were performed in accordance with relevant guidelines and regulations.

Only variables that were available before the start time of the surgical procedure were used as features to predict intraoperative allogeneic red blood cell (RBC) requirements. For example, a prior surgical procedure could be used as one, among several, important features to predict transfusions for the upcoming surgery. Missing values of categorical features for a given patient indicated that feature was not present. Missing values of continuous features for a given patient were transformed to the median value in the dataset for that particular feature. After a discussion with cardiothoracic (CT) surgeon stakeholders about perceived value, we decided a priori to attempt to predict intraoperative blood use based on the following usage categories: 0 units, 1–3 units, and 4 + units. The value of an accurate prediction is to facilitate appropriate blood ordering practices beyond a maximum surgical blood order schedule (MSBOS) and to identify cases that are likely to experience significant hemorrhage.

**Figure 1:**
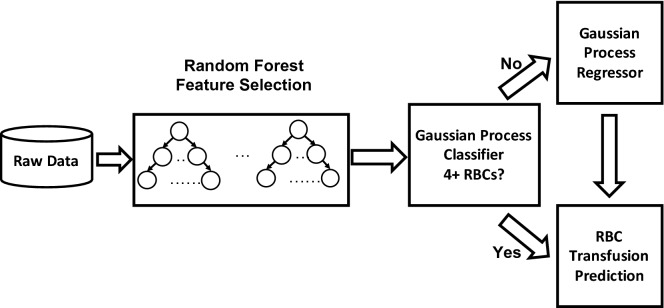
Feature selection and hybrid machine learning (ML) model. The raw cardiothoracic (CT) surgery dataset contained numerous features. To reduce the features to a manageable number, a random forest feature selection procedure was performed. The selected features were then used as inputs into the Gaussian Process (GP) regression and classification ML algorithms.

To build a competent model that can be adequately explained, we adopt a hybrid machine learning (ML) framework. Instead of feeding all features into our algorithm, we started with a random forest (RF) algorithm to filter out unimportant features (Fig. 1). We chose RF for feature selection because we assumed nonlinear relationships between the inputs and the outputs to improve accuracy. RF is a widely used ensemble method in ML that can effectively reduce prediction variance by averaging predictions across many randomly generated decision trees. For a decision tree, the split for each node is determined by impurity reduction which is typically measured by entropy, or gini index, and the resulting structure can resemble human reasoning. RFs can compute feature importance by averaging the probability of reaching a certain node through the corresponding feature. Thus, with feature importance, a filtering threshold can be set to select only strongly associated features. Note that popular feature selection methods, e.g., lasso and elastic net, are based on a linear regression model, which assume all the features are linearly correlated to the outcome. We did consider these commonly used linear feature selection methods, but these demonstrated inferior performance. By contrast, our RF feature selection approach does not restrict to linear correlations as it also covers strong nonlinear correlations, which can facilitate the prediction task in the next step.

After the initial feature selection procedure, we imposed a Gaussian process (GP) regression model to capture the complex relationships between the clinical data and intraoperative RBC transfusion amount. We chose GP for two reasons. First, GP is a nonparametric model, can self-adapt the model complexity (e.g. linear to highly nonlinear) according to the data, and hence alleviates both underfitting and overfitting (the latter is a disadvantage of neural networks). Second, the posterior distribution of GP has a closed-form, and hence the uncertainty quantification is convenient. GP is also well known for the fact that neural networks can be turned into a GP model under certain conditions, which translates to the ability to capture complex nonlinear relationships. GP is proposed to learn a mapping or function $f: {\mathbb{R}}^{d}\to {\mathbb{R}}$ from a training set $D=\left(\mathbf{X}, \mathbf{y}\right)$, where $\mathbf{X}={\left[{\mathbf{x}}_{1}, \dots , {\mathbf{x}}_{N}\right]}^{\mathrm{T}}$, $\mathbf{y}={[{y}_{1}, \dots , {y}_{N}]}^{\mathrm{T}}$, each ${\mathbf{x}}_{N}$ is a $d$ dimensional input vector, i.e. selected clinical data features, and ${y}_{n}$ the observed output, i.e. the amount of RBC components transfused. Usually, the GP model associated with $f$ is denoted as $f\sim GP(m\left(\cdot \right), k\left(\cdot , \cdot \right))$, where $m\left(\cdot \right)$ is the mean function, often set to be 0, and $k\left(\cdot ,\cdot \right)$ is the covariance (kernel) function. With $\mathbf{f}={[f\left({\mathbf{x}}_{1}\right),\dots ,f({\mathbf{x}}_{N})]}^{\mathrm{T}}$ following a multivarate Gaussian distribution $N(\mathbf{f}|0, \mathbf{K})$, where $\mathbf{K}$ is the kernel matrix on $\mathbf{X}$, and an isotropic Gaussian noise, the conditional marginal distribution can be derived as $p\left(\mathbf{y}|\mathbf{X}\right)=N(\mathbf{y}|0, \mathbf{K}+{\tau }^{-1}\mathbf{I})$, where $\tau$ is the inverse variance of the noise. The kernel parameters and $\tau$ are parameters to learn by maximizing the likelihood of the data. The prediction can be obtained via the posterior distribution, which is a conditional Gaussian distribution. We compared our GP regression model performance to another well-known approach, a random forest regression ensemble model.

Finally, to account for the highly skewed distribution of higher blood use surgical cases (4 + units), we transformed the original problem into a binary classification task because regression based on highly skewed class distribution can result in poor performance. We compared the performance of GP classification to other well-known machine learning classification algorithms, including neural networks, XGBoost, random forest, and decision tree. Both the regression and classification machine learning algorithms used to train the data employed a five-fold crossvalidation approach, which is a standard approach to reduce overfitting.

To validate the model performance at our institution, we expanded the dataset to include all CT surgery cases occurring from August 2019 through August 2020. An algorithm required by our EDW to reduce the risk of a data breach included random forward date shifting from 1 to 90 days after the true date for all time-stamped variables. If dates were shifted forward a given number of days, this applied to all time-stamped variables for a given case. We elected to exclude the first three months of data from the validation dataset to eliminate the chance of overlap between the training and validation datasets. The original dataset’s cases were then used as a training dataset and the additional cases included after updating the dataset were used as a test dataset for validation of all previously mentioned ML algorithms.

## Results

### Patient characteristics

The initial training dataset included 2410 Cardiothoracic (CT) surgery patients from May 2014 to July 2019. The validation dataset included an additional 437 CT surgery patients from August 2019 to August 2020. Therefore, the total number of cases included in the development of our optimized machine learning (ML) algorithm was 2847. Detailed information about the characteristics of included patients is shown in Table 1. This includes demographics, blood utilization, common laboratory values, comorbidities existing prior to surgery, and the most common procedure types. Most patients received zero intraoperative allogeneic RBC transfusions, while about one quarter used 1–3 units and 6% used 4 or more units. This right-skewed distribution is typical for plots showing the frequency of transfusion volumes amongst a cohort of patients^18,19^. While most patients were male, the percentage of female patients increased in higher blood use categories. The hemoglobin value before surgery was lower in higher blood use categories.

**Table 1:** Combined characteristics of cases in the training (development) and test (validation) datasets.

	All patients (test + training datasets)	0 units transfused	1–3 units transfused	4 + units transfused
Number of cases	2847 (100%)	1962 (69%)	712 (25%)	173 (6%)
Age (years)	58.9	58.4	60.0	58.9
Gender (% female)	31	27	39	42
**Ethnicity (%)**				
Not Hispanic/Latino	89	89	88	86
Hispanic/Latino	7	7	7	6
Unknown	4	4	5	8
**Race**				
White/Caucasian	83	85	78	79
Other	6	6	7	6
Black or African American	3	3	5	4
American Indian and Alaska Native	3	2	4	2
Unknown	3	2	2	5
Asian	1	1	2	2
Native Hawaiian and Other Pacific Islander	1	1	2	2
RBC transfusions (mean)	1.26	0	1.62	6.34
Plasma transfusions (mean)	1.36	0.73	1.83	6.58
Platelet transfusions (mean)	0.66	0.34	1.17	2.18
Hemoglobin before surgery	12.15	13.08	10.45	10.25
Hemoglobin after surgery	9.61	9.91	8.96	8.87
Platelet count before surgery	204	211	193	175
Creatinine before surgery	1.41	1.28	1.63	1.69
Cell salvage used (ml)	443	433	381	817
Pre-existing end stage renal disease	145/2847 (5%)	53/1962 (3%)	71/712 (10%)	21/173 (12%)
Pre-existing hypertension	1647/2847 (58%)	1094/1962 (56%)	426/712 (60%)	127/173 (73%)
Pre-existing peripheral artery disease	96/2847 (3%)	53/1962 (3%)	36/712 (5%)	7/173 (4%)
Pre-existing cerebrovascular disease	132/2847 (5%)	68/1962 (3%)	50/712 (7%)	14/173 (8%)
Pre-existing diabetes mellitus	705/2847 (25%)	444/1962 (23%)	215/712 (30%)	46/173 (27%)
**Case type/urgency (%)**				
Elective	73	80	61	46
Urgent	17	15	24	22
Emergent	10	5	15	32
**Most common procedures (%)**				
	CABG (26)	CABG (31)	CABG (15)	Ascending aortic dissection (13)
	Placement LVAD (7)	Placement LVAD (7)	Mediastinal exploration (10)	Transplant heart (10)
	Transplant heart (5)	Replacement aortic valve (5)	Placement LVAD (8)	Placement LVAD (8)
	Mediastinal exploration (4)	CABG with aortic valve replacement (3)	Transplant heart (8)	Aortic valve and mitral valve replacement (5)
	Replacement aortic valve (4)	Transplant heart (3)	Replacement aortic valve (3)	Mediastinal exploration (4)
	CABG with aortic valve replacement (3)	Mitral valve replacement (3)	Ascending aortic aneurysm (3)	CABG (8)
	Mitral valve replacement (3)	Minimally invasive aortic valve replacement (3)	CABG with aortic valve replacement (3)	Sternal exploration (4)
	Ascending aortic dissection (3)	Ascending aortic aneurysm repair (3)	Placement ECMO (3)	Thoracoabdominal aortic aneurysm repair (4)
	Ascending aortic aneurysm repair (2)	Mediastinal exploration (3)	Transplant double lung with bypass (3)	Placement ECMO (3)

*CABG* coronary artery bypass graft, *LVAD* left ventricular assist device, *ECMO* extracorporeal membrane oxygenation.

### Feature selection

The dataset was wide, containing a large number of variables per patient. The initial number of features in the dataset was 10,622. The feature selection procedure used random forest to reduce the number of features down to 202 based on the importance value of each feature. Features were considered important if they were involved in a node split that usefully divided the dataset into different groups based on transfusion amount. Table 2 shows the top 10 selected features, which is a subset of the 202 total features selected (Supplementary Table S2 includes a list of all 202 selected features). The importance value reflects the percentage of the time a given feature was involved in usefully dividing the data in the decision trees that comprised the random forest. The features remaining after the feature selection procedure were then used to train the different ML algorithms tested.

**Table 2:** Top 10 features after random forest feature selection.

Rank	Feature	Feature description^a^	Importance	Number of cases where feature was present/total cases used for feature selection
1	CPT code	Veno-arterial extracorporeal membrane oxygenation (ECMO) initiation	0.020874491	114/2410
2	ICD-10 procedure code	ECMO continuous	0.015373679	56/2410
3	CPT code	ECMO cannulation	0.013020617	94/2410
4	CPT code	Thoracoabdominal aortic aneurysm repair	0.010975536	10/2410
5	Laboratory result	Blood gas analysis, barometric pressure	0.010827222	863/2410
6	Laboratory result	Blood gas analysis, potassium	0.009008439	843/2410
7	Laboratory result	Ionized calcium	0.00858092	945/2410
8	Laboratory result	Hemoglobin	0.007877384	2125/2410
9	Laboratory result	Albumin	0.00751122	1756/2410
10	ICD-10 code	Respiratory ventilation > 96 h	0.007166464	113/2410

*CPT* current procedural terminology, *ICD* international classification of diseases.

^a^All features used were only applied if they were known to be available for the patient prior to the surgery start time. Features (e.g. laboratory values, billing codes) were excluded from the model for a given patient if they were not available before the surgery start time.

### Predicting intraoperative RBC utilization using regression

We report the regression results in Table 3 using root mean square error (RMSE) to evaluate the deviation of predictions from the true values. Aside from our Gaussian process (GP) method, we also tested random forest and all results are obtained by splitting the data into five training and testing folds with mean value and standard deviation. For less severe cases (RBC usage less than or equal to 3 units), our regression method showed excellent prediction performance in both the development and validation phases as indicated by the low RMSE values (Table 3). For more severe cases (RBC usage 4 or more units), performance of both methods declined, likely due to the highly skewed nature of the data (Table 3).

**Table 3:** Random Forest (RF) versus Gaussian process (GP) regression.

	0 units transfused	1–3 units transfused	4 + units transfused
**Model development**			
Random forest	0.829 (0.043)	1.191 (0.047)	5.799 (0.612)
Gaussian process regression	0.064 (0.102)	1.758 (0.033)	7.613 (0.635)
Gaussian process regression for less severe cases	0.766 (0.016)		
**Model validation**			
Random forest	7.007	1.624	56.568
Gaussian process regression	0.117	1.705	56.941
Gaussian process regression for less severe cases	0.985		

This table shows our GP regression model compared with the RF regression model. GP regression performed best as demonstrated by the low root mean square error (RMSE) in the 0 units transfused and 1–3 units transfused categories. In contrast, performance of both models suffered when predicting 4 + RBCs transfused. Therefore, in the final model we restricted the GP regression prediction to cases where < 4 RBCs transfused was predicted by GP classification.

### Classification algorithm for prediction of 4 + units transfused

**Figure 2:**
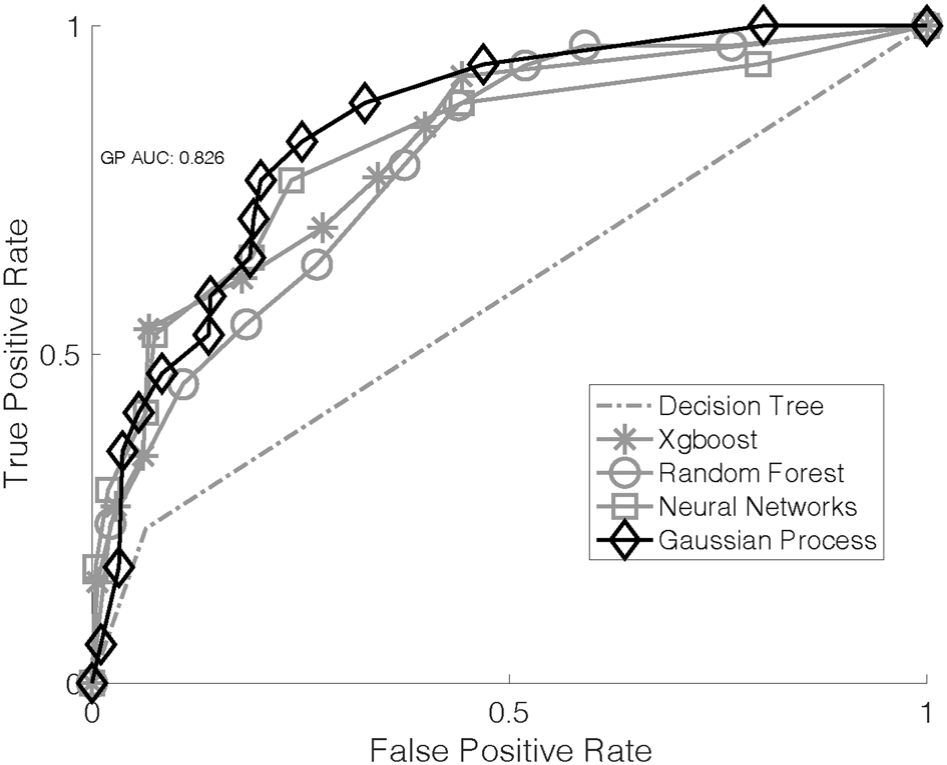
Area under the curve (AUC) for each machine learning (ML) classification algorithm for the development phase. Here, we used only the initial dataset (n = 2410) with five-fold crossvalidation to reduce overfitting. Gaussian Process (GP) demonstrated the greatest AUC and overall performance.

**Figure 3:**
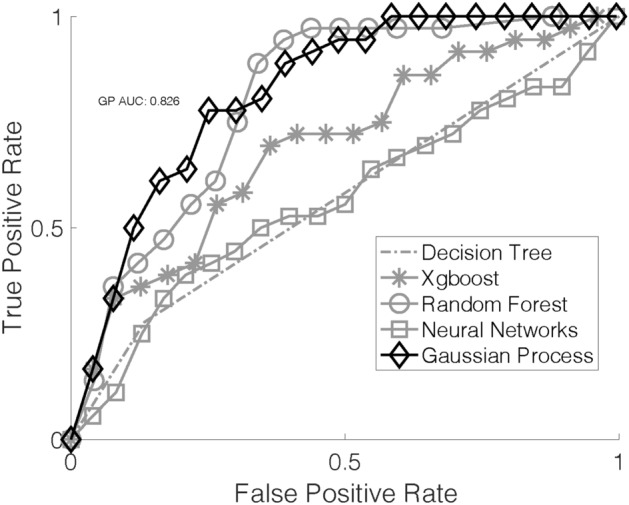
Area under the curve (AUC) for each machine learning (ML) classification algorithm after the validation phase. Here, the initial dataset (n = 2410) was used as the training dataset and the additional cases included after updating the database (n = 437) were used as the test dataset. Gaussian Process (GP) again showed the highest AUC and best overall performance.

To evaluate the discriminative performance of our method in both the development and validation phases, we use area under the receiver operator curve (AUC) that summarizes the trade-off between sensitivity (true positive) and specificity (true negative). We also tested some other methods by replacing the GP classifier with a boosting classifier, decision trees, or neural networks. Here, we directly utilize XGboost, a widely used and powerful library for the boosting algorithm, and run the boosting classifier. In addition to AUC, we also report sensitivity and specificity at the cut-off point of the curve, selected as the intersection point of the AUC curve and the line $y=-x+1$, and the F1 score (harmonic mean of the sensitivity and positive predictive value for severe case classification). Our GP classification method demonstrated the best overall performance in the development phase (AUC = 0.80) and its performance improved in the validation phase with an AUC value of 0.82 (Table 4, Figs. 2 and 3). Random forest, decision tree, and XGboost models demonstrated good specificity, but their sensitivity and F1 scores were low (Table 4).

**Table 4:** 4 + RBCs transfused classification.

	AUC	Sensitivity	Specificity	F1 score
**Model development**				
Gaussian process	0.826 (0.017)	0.892 (0.027)	0.678 (0.030)	0.766 (0.015)
Random forest	0.812 (0.009)	0.812 (0.052)	0.668 (0.052)	0.726 (0.009)
Decision tree	0.610 (0.021)	0.272 (0.044)	0.948 (0.007)	0.413 (0.055)
XGBoost	0.820 (0.015)	0.805 (0.046)	0.741 (0.051)	0.757 (0.013)
Neural network	0.723 (0.027)	0.682 (0.055)	0.742 (0.037)	0.697 (0.025)
**Model validation**				
Gaussian process	0.826	0.778	0.771	0.774
Random forest	0.803	0.944	0.642	0.764
Decision tree	0.572	0.278	0.866	0.421
XGBoost	0.697	0.694	0.659	0.676
Neural network	0.760	0.833	0.679	0.748

Multiple machine learning (ML) models were compared using several performance metrics. Our Gaussian Process (GP) classification model demonstrated the best performance in the development phase and in the subsequent validation phase. *AUC* area under the receiver operator curve. F1 Score = the harmonic mean of the sensitivity and positive predictive value. Note that standard deviations are only listed for the model development phase that used the initial dataset because it used five-fold cross validation. The model validation phase used the initial dataset for training (n = 2410) and the additional set of cases (n = 437) for its test phase.

The final hybrid ML algorithm incorporated GP classification and, conditionally, GP regression. If the GP classification result was < 4 RBCs transfused, then GP regression was used to predict the specific amount of RBCs transfused. If the GP classification prediction was > 4 RBCs, then GP regression was not used. We propose this approach to maximize the practical value of the prediction while still accounting for the skewed nature of the dataset.

## Discussion

Blood utilization in cardiothoracic (CT) surgery varies, with some cases requiring no blood transfusions while others may require massive transfusion. The ability to predict intraoperative RBC utilization with high accuracy could serve a dual function: informing risk of significant hemorrhage as well as optimal blood ordering and crossmatching. We developed a machine learning (ML) approach to address this problem using inputs from a detailed clinical database at our institution. Our approach showed excellent performance that was subsequently validated with an updated dataset. Our database containing model inputs was local and wide (i.e. contained numerous variables per patient). We first used a feature selection procedure to scale down the number of model inputs followed by application of several ML models for prediction performance comparisons. Our final hybrid Gaussian Process (GP) ML model outperformed the other common ML algorithms that we tested. To our knowledge, this is the first ML-based approach for predicting RBC transfusions specifically in CT surgery.

We found that a regression approach to prediction resulted in excellent performance when predicting zero units transfused as well as 1–3 units transfused. This approach did not perform well when predicting higher blood use cases (4 + units), which was demonstrated by the relatively high root mean square error (RMSE) for this usage category. This is most likely due to there being relatively fewer events in the 4 + units transfused category (173 total) when compared with the other categories as well as there being a larger range of RBCs transfused within that category. For this reason, we viewed the higher RBC use prediction as a classification problem. GP classification resulted in strong performance with an AUC of 0.82. With the initial dataset, we used five-fold cross-validation for all ML models tested, which is a standard approach to reduce overfitting. Our GP classification model outperformed the other classification models we tested, which included simpler (decision tree) and more complex/black box models (random forest, XGBoost, and neural networks). Therefore, the ultimate approach we developed includes feature selection using random forest followed by a hybrid ML approach with GP classification and, if fewer than 4 RBCs transfused was predicted, GP regression.

After we developed the hybrid ML approach using the original dataset (May 2014–June 2019), we validated our model. We updated the clinical database to include cases up through August 2020, an additional 437 cases. Here, we trained the GP algorithms using the original 2140 patient dataset and next tested the GP algorithms with the new 437 cases. The model performance remained strong and even improved for the classification prediction of 4 + RBCs transfused. The slight improvement in predicting these larger transfusion volumes may be due to the increased number of cases from which we could train the GP classification algorithm.

Maximum surgical blood order schedules (MSBOS) are a well-known method for institutions to standardize blood ordering prior to major surgery^9^. While MSBOSs provide value by improving blood inventory management and limiting excessive ordering, they do not provide a personalized prediction of blood utilization and associated hemorrhage risk. Beyond MSBOSs, multiple prior statistical models predicting transfusions in cardiac surgery have been developed, but to our knowledge none has been widely adopted^4–8^. These prior models all used a classification approach, but there were notable differences in their methods. Some attempted to predict zero versus any transfusion, while others attempted to predict massive transfusion. Further, the period of which blood use was predicted was often well after the surgery, sometimes for the entire hospital stay. Some included variables that could only be obtained after a surgery had started, further limiting the value of the prediction. It is worth noting that while our ML approach was not based on making a priori assumptions, our model did ultimately include features that had been shown to be predictive in the past (examples include age, laboratory values associated with anemia, history of major prior surgeries, and more)^4–8^. Our ML-based approach improves upon prior models by accounting for complex nonlinear relationships of predictive features, using only preoperative variables, predicting a broader range of outcomes (from zero to 4 + units transfused), and restricting our prediction to intraoperative RBCs transfused.

It is important to note that the best approach we identified in this study was a black box model (Gaussian Process, GP). This and other black box models (XGBoost, random forest, neural networks) outperformed the much simpler and more transparent decision tree. The advantage of a simpler model is easier clinical interpretation due to transparency. For example, if a small number of features were sufficiently predictive (e.g. age, preoperative hemoglobin, surgeon, anesthesiologist, procedure performed, preoperative creatinine), these predictors might make intuitive sense to clinicians and provide initial confidence in the model. However, we found that decision tree performed poorly.

Recommendations for how to develop and present ML models in healthcare are available^20,21^. While we incorporated recommended items for developing and validating a prediction model, it is important to note that our study has some unique characteristics. First, our ultimate ML model is “black box” as noted above. Second, we used a large number of features for prediction, even after feature selection. While we achieved a reasonable sample size using local data (n = 2847), the number of features is still considered high. We would like to emphasize that the generalizability of this study is in the prediction method, rather than in the prediction model itself. This has been described as a new approach wherein models are intentionally trained and re-trained over time using local data^21^. We use this approach here because we assumed the degree of hemorrhage and need for blood transfusions in cardiothoracic surgery is inherently complex and likely cannot be explained adequately by simpler, more transparent models. This is supported by the poor performance of the decision tree model in our study. For other institutions to apply our approach, we suggest developing a detailed clinical database using local data, performing feature selection (e.g. random forest), and evaluating our hybrid GP classification and regression method performance using their data. This could be validated on local data and also compared to other commonly used ML models, similar to our approach.

The use of ML for prediction in healthcare is growing as development and availability of detailed clinical databases allows investigators to predict any outcome of interest. While there is enthusiasm surrounding ML, its value in healthcare remains to be seen in many cases. ML has a better chance to provide value in a given context when a systematic approach to conception, development, and validation is taken^10^. Our aim with this study was to take the first key step of developing and validating a blood use prediction model in a highly relevant (commonly transfused) patient population at our institution.

Strengths of this study include the systematic approach to database development and validation with our Enterprise Data Warehouse (EDW). We compared several well-known ML models to each other and used a five-fold cross-validation approach for model development. We expanded the dataset to include additional patients and the models continued to demonstrate excellent performance.

This study also has limitations. ML prediction models always have a risk of overfitting, particularly with a single-center dataset that, by nature, limits the number of cases for analysis. We sought to mitigate this overfitting risk using five-fold cross-validation initially, followed by validation with a separate dataset of subsequent patients from our institution. This study also notably used data from our local Enterprise Data Warehouse (EDW). EDW datasets can be updated regularly at short intervals, but implementation into practice may require that some features (e.g. lab values) are extracted from the electronic health record (EHR) in real time if EDW datasets cannot update with sufficient speed. Last, we focused on predicting intraoperative RBC transfusions and did not evaluate other blood components, such as plasma, platelets, or cryoprecipitate.

Future directions include further validation of performance of our ML prediction method within our institution and at other institutions. A stable, validated model using local data could then be carefully implemented for routine use by surgeons and anesthesiologists. Implementation may take the form of an electronic health record (EHR) application that is ultimately integrated into the routine clinical workflow for preoperative blood ordering. We envision this application would perform all necessary steps to achieve an accurate prediction so that the end user is given a straightforward prediction result. The application would use our selected features to first perform GP classification in the background. If 4 + RBCs predicted, this would be the final prediction presented to the end user. If 0–3 RBCs predicted, then GP regression would be performed and this more specific regression result would be presented to the end user, rounded to the nearest integer. The end user could use this information to help decide whether and how much blood should be ordered for the upcoming surgery and to evaluate hemorrhage risk.

## Conclusion

In conclusion, we developed and validated an ML-based method to predict intraoperative RBC transfusion requirements in CT surgery using local data. Our method demonstrated excellent performance, but requires further validation outside our institution.

## Supplementary Information

Supplementary Table 1.

Supplementary Table 2.
