# Supplementary material for: Development and validation of a machine learning method to predict intraoperative red blood cell transfusions in cardiothoracic surgery
Source: Sci Rep. 2022 Jan 25;12:1355. doi: 10.1038/s41598-022-05445-y (PMC8789772; doi:10.1038/s41598-022-05445-y)
Supplement: Supplementary file 1 — Supplementary Table 1. [file 41598_2022_5445_MOESM1_ESM.docx]

**Supplemental Table 1** : The table below provides an idea of the variables included in the cardiothoracic (CT) surgery database, such as demographics, visit information, surgery case information, billing codes, and much more. Data that occurred during or after the CT surgery case were excluded as predictive features.

1. CLIN_DM.BPU_CTS_DI_PATIENT 4633

DI_PAT_ID NUMBER(38)

DI_BIRTHDATE DATE

GENDER_CODE VARCHAR2(80)

GENDER_DESC VARCHAR2(2000)

RACE_CODE VARCHAR2(80)

RACE_DESC VARCHAR2(2000)

ETHNICITY_CODE VARCHAR2(80)

ETHNICITY_DESC VARCHAR2(2000)

DI_DEATH_DATE DATE

2. CLIN_DM.BPU_CTS_DI_VISIT 4623

DI_PAT_ID NUMBER(38)

DI_VISIT_NO NUMBER(38)

DI_ADM_DTM DATE

DI_DSCH_DTM DATE

AGE_AT_ADM NUMBER

PAT_CLASS_DESC VARCHAR2(2000)

PAT_TYPE_DESC VARCHAR2(2000)

PAT_EXPIRED CHAR(1)

INVASIVE_VENT_F CHAR(1)

TOTAL_VENT_MINS NUMBER

TOTAL_VENT_DAYS NUMBER

APR_DRG_CODE VARCHAR2(254)

APR_DRG_ROM VARCHAR2(80)

APR_DRG_SOI VARCHAR2(80)

APR_DRG_DESC VARCHAR2(200 CHAR)

APR_DRG_WEIGHT NUMBER

3. CLIN_DM.BPU_CTS_DI_SURGERY_CASE 4623

DI_PAT_ID NUMBER(38)

DI_VISIT_NO NUMBER(38)

DI_CASE_ID NUMBER(38)

DI_CASE_DATE DATE

DI_SURGERY_START_DTM DATE

DI_SURGERY_END_DTM DATE

SURGERY_ELAP NUMBER

SURGERY_TYPE_DESC VARCHAR2(2000)

SURGEON_PROV_DWID NUMBER(38)

ANESTH_PROV_DWID NUMBER(38)

PRIM_PROC_DESC VARCHAR2(2000)

POSTOP_ICU_LOS NUMBER

SCHED_SITE_DESC VARCHAR2(2000)

4. CLIN_DM.BPU_CTS_DI_BILLING_CODES 1104033

DI_PAT_ID NUMBER(38)

DI_VISIT_NO NUMBER(38)

DI_ADM_DTM DATE

DI_DSCH_DTM DATE

CODE_SEQNUM NOT NULL NUMBER

CODE_TYPE_DESC VARCHAR2(2000)

CODE_VERSION_DESC VARCHAR2(2000)

CODE VARCHAR2(80)

CODE_DESC VARCHAR2(2000)

DI_PROC_DTM DATE

PROV_DWID NUMBER(38)

PRESENT_ON_ADM_F CHAR(1)

5. CLIN_DM.BPU_CTS_DI_VST_LABS 12040302

DI_PAT_ID NUMBER(38)

DI_VISIT_NO NUMBER(38)

DI_ADM_DTM DATE

DI_DSCH_DTM DATE

DI_DRAW_DTM DATE

LAB_PANEL_CODE VARCHAR2(30)

LAB_PANEL_DESC VARCHAR2(256)

DI_RESULT_DTM DATE

RESULT_CODE VARCHAR2(30)

RESULT_LOINC VARCHAR2(30)

RESULT_DESC VARCHAR2(256)

RESULT_VALUE VARCHAR2(1000)

UOM_CODE VARCHAR2(30)

UOM_TEXT VARCHAR2(256)

REFERENCE_RANGES VARCHAR2(256)

REF_LOWER_LIMIT NUMBER

REF_UPPER_LIMIT NUMBER

6. CLIN_DM.BPU_CTS_DI_EXTRAOP_VITALS 1696438

DI_PAT_ID NUMBER(38)

DI_VISIT_NO NUMBER(38)

DI_ADM_DTM DATE

DI_DSCH_DTM DATE

DI_OBS_DTM DATE

TEMP_C NUMBER

BP_SYST NUMBER

BP_DIAST NUMBER

BP_MAP NUMBER

HEART_RATE NUMBER

RESP_RATE NUMBER

SPO2 NUMBER

BMI NUMBER

BSA NUMBER

HEIGHT_CM NUMBER

WEIGHT_KG NUMBER

7. CLIN_DM.BPU_CTS_DI_INTRAOP_MEDS 140829

DI_PAT_ID NUMBER(38)

DI_VISIT_NO NUMBER(38)

DI_ADM_DTM DATE

DI_DSCH_DTM DATE

DI_CASE_ID NUMBER(38)

DI_ORDER_DTM DATE

MEDICATION_ID NUMBER(18)

MEDICATION_NAME VARCHAR2(510)

DI_ADMIN_DTM DATE

ADMIN_DOSE VARCHAR2(184)

MED_FORM VARCHAR2(50)

ADMIN_ROUTE_DESC VARCHAR2(254)

DOSE_UNIT_DESC VARCHAR2(254)

DI_MED_START_DTM DATE

DI_MED_END_DTM DATE

8. CLIN_DM.BPU_CTS_DI_EXTRAOP_MEDS 5534097

DI_PAT_ID NUMBER(38)

DI_VISIT_NO NUMBER(38)

DI_ADM_DTM DATE

DI_DSCH_DTM DATE

DI_ORDER_DTM DATE

MEDICATION_ID NUMBER(18)

MEDICATION_NAME VARCHAR2(510)

DI_ADMIN_DTM DATE

ADMIN_DOSE VARCHAR2(184)

DOSE_UNIT_DESC VARCHAR2(254)

MED_FORM VARCHAR2(50)

ADMIN_ROUTE_DESC VARCHAR2(254)

DI_MED_START_DTM DATE

DI_MED_END_DTM DATE

9. CLIN_DM.BPU_CTS_DI_BLD_PROD_ISSUED 76054

DI_PAT_ID NUMBER(38)

DI_VISIT_NO NUMBER(38)

DI_ADM_DTM DATE

DI_DSCH_DTM DATE

DI_PRODINV_ID NUMBER(38)

STANDARD_PRODUCT_CODE VARCHAR2(255)

PRODUCT_ID VARCHAR2(255)

DI_DRAW_DATE DATE

DI_EXPIRATION_DATE DATE

DI_ISSUE_DTM DATE

ISSUE_TO_LOC_ID VARCHAR2(255)

ISSUE_TO_SUBLOC_ID VARCHAR2(255)

INVENTORY_STATUS_CD VARCHAR2(255)

INVENTORY_STATUS_DESC VARCHAR2(255)

DI_ORD_PROV_ID NUMBER(38)

AVAILABLE_QUANTITY VARCHAR2(255)

PROD_ABO VARCHAR2(255)

PROD_RH VARCHAR2(255)

NON_EMERGENT_F CHAR(1)

EMERGENT_F VARCHAR2(1)

NON_OR_UNIT_F CHAR(1)

OR_UNIT_F CHAR(1)

CROSSMATCHED_F CHAR(1)

TRANSFUSED_F CHAR(1)

RETURNED_F CHAR(1)

RETURN_DISP VARCHAR2(255)

10. CLIN_DM.BPU_CTS_DI_BLD_PROD_CMTCH 4338

DI_PAT_ID NUMBER(38)

DI_VISIT_NO NUMBER(38)

DI_CMPLTN_DATE DATE

DI_PRODINV_ID NUMBER(38)

PRODUCT_ID VARCHAR2(255)

TEST_ID VARCHAR2(255)

TEST_DESC VARCHAR2(255)

DI_ORD_PROV_ID NUMBER(38)

PROD_ABO VARCHAR2(255)

PROD_RH VARCHAR2(255)

11 CLIN_DM.BPU_CTS_DI_INTRAOP_TRNSFSD 13512

DI_PAT_ID NUMBER(38)

DI_VISIT_NO NUMBER(38)

DI_CASE_ID NUMBER(38)

DI_CASE_DATE DATE

DI_SURGERY_START_DTM DATE

DI_SURGERY_END_DTM DATE

DI_TRNSFSN_DTM DATE

PRBC_UNITS VARCHAR2(2500)

FFP_UNITS VARCHAR2(2500)

PLT_UNITS VARCHAR2(2500)

CRYO_UNITS VARCHAR2(2500)

CELL_SAVER_ML VARCHAR2(2500)

12 CLIN_DM.BPU_CTS_DI_EXTRAOP_TRNSFSD 78662

DI_PAT_ID NUMBER(38)

DI_VISIT_NO NUMBER(38)

DI_ADM_DTM DATE

DI_DSCH_DTM DATE

DI_ORDER_DTM DATE

PROC_ID NUMBER(18)

PROC_DESC VARCHAR2(254)

ORDER_UNIT_DESC VARCHAR2(2000)

PRODUCT_CODE VARCHAR2(50)

PRODUCT_ID VARCHAR2(4)

DI_PROC_START_DTM DATE

DI_PROC_END_DTM DATE

DI_VOL_RECORD_DTM DATE

TRNSFSN_VOL_ML VARCHAR2(2500)

13 CLIN_DM.BPU_CTS_DI_PREOP_LABS 12527626

DI_PAT_ID NUMBER(38)

DI_VISIT_NO NUMBER(38)

DI_ADM_DTM DATE

DI_DSCH_DTM DATE

DI_DRAW_DTM DATE

LAB_PANEL_CODE VARCHAR2(30)

LAB_PANEL_DESC VARCHAR2(256)

DI_RESULT_DTM DATE

RESULT_CODE VARCHAR2(30)

RESULT_LOINC VARCHAR2(30)

RESULT_DESC VARCHAR2(256)

RESULT_VALUE VARCHAR2(1000)

UOM_CODE VARCHAR2(30)

UOM_TEXT VARCHAR2(256)

REFERENCE_RANGES VARCHAR2(256)

REF_LOWER_LIMIT NUMBER

REF_UPPER_LIMIT NUMBER
