# Supplementary material for: Development and validation of a machine learning method to predict intraoperative red blood cell transfusions in cardiothoracic surgery
Source: Sci Rep. 2022 Jan 25;12:1355. doi: 10.1038/s41598-022-05445-y (PMC8789772; doi:10.1038/s41598-022-05445-y)
Supplement: Supplementary file 2 — Supplementary Table 2. [file 41598_2022_5445_MOESM2_ESM.docx]

**Supplementary Table 2: List of Features After Random Forest Feature Selection**

This table includes a list of the 202 remaining features after random forest feature selection. These remaining features were used for prediction of intraoperative red blood cells transfused, where the models with the best performance after development and validation phases were Gaussian Process classification and regression.

| FEATURE RANK | FEATURE CODE | FEATURE DESCRIPTION | FEATURE IMPORTANCE |
| --- | --- | --- | --- |
| 1 | CODE_33947 | ECMO/ECLS INITIATION VENO-ARTERIAL | 0.020874491 |
| 2 | CODE_5A15223 | EXTRACORPOREAL MEMBRANE OXYGENATION CONTINUOUS | 0.015373679 |
| 3 | CODE_33956 | ECMO/ECLS INSJ OF CENTRAL CANNULA 6 YRS & OLDER | 0.013020617 |
| 4 | CODE_33877 | RPR THORACOABDOMINAL AORTIC ANEURYS W/WO BYPASS | 0.010975536 |
| 5 | RESULT_22 | BLOOD GAS ANALYSIS WITH ELECTROLYTES (AEGIS)--Pb | 0.010827222 |
| 6 | RESULT_26 | BLOOD GAS ANALYSIS WITH ELECTROLYTES (AEGIS)--K+ | 0.009008439 |
| 7 | RESULT_34 | ASSAY OF CALCIUM, IONIZED--Temp | 0.00858092 |
| 8 | RESULT_40085 | CBC with Plt Count and Auto Diff--Hemoglobin | 0.007877384 |
| 9 | RESULT_20030 | Comprehensive Metabolic Panel--Albumin, Serum or Plasma | 0.00751122 |
| 10 | CODE_5A1955Z | RESPIRATORY VENTILATION > 96 CONSECUTIVE HOURS | 0.007166464 |
| 11 | CODE_32505 | THORACOTOMY W/THERAPEUTIC WEDGE RESEXN INITIAL | 0.006553255 |
| 12 | CODE_04R00JZ | REPLACEMENT ABD AORTA W/SYNTH SUBST OPEN APPRCH | 0.006550716 |
| 13 | RESULT_70133 | Triiodothyronine, Free (Free T3)--Triiodothyronine, Free (Free T3) | 0.006516891 |
| 14 | RESULT_40080 | CBC with Plt Count and Auto Diff--Hematocrit | 0.006492949 |
| 15 | RESULT_40270 | CBC with Platelet Count--Red Blood Cell | 0.006141843 |
| 16 | RESULT_14 | BLOOD GAS ANALYSIS WITH ELECTROLYTES (AEGIS)--pH | 0.006097937 |
| 17 | CODE_82962 | GLUC BLD GLUC MNTR DEV CLEARED FDA SPEC HOME USE | 0.006013701 |
| 18 | CODE_33875 | DESCENDING THORACIC AORTA GRAFT W/WO BYPASS | 0.005928886 |
| 19 | CODE_95939 | CTR MOTR EP STD TRANSCRNL MOTR STIM UPR&LOW LI | 0.005715747 |
| 20 | CODE_5A1522F | EXTRACORPOREAL OXYGENATION, MEMBRANE, CENTRAL | 0.005653803 |
| 21 | RESULT_15 | BLOOD GAS ANALYSIS WITH ELECTROLYTES (AEGIS)--PaCO2 | 0.005599613 |
| 22 | RESULT_40180 | CBC with Platelet Count--Mean Platelet Volume | 0.005570479 |
| 23 | RESULT_2013434 | CBC with Plt Count and Auto Diff--Immature Granulocytes % | 0.005467679 |
| 24 | CODE_93325 | DOP ECHOCARD COLOR FLOW VELOCITY MAPPING | 0.005194919 |
| 25 | RESULT_40061 | CBC with Plt Count and Auto Diff--Eosinophil % | 0.004863058 |
| 26 | RESULT_17 | BLOOD GAS ANALYSIS WITH ELECTROLYTES (AEGIS)--O2Hb | 0.004712823 |
| 27 | CODE_0KNT0ZZ | RELEASE LEFT LOWER LEG MUSCLE OPEN APPROACH | 0.004634481 |
| 28 | RESULT_20396 | Basic Metabolic Panel--Anion Gap | 0.004450825 |
| 29 | RESULT_20006 | Lactate Dehydrogenase, Plasma or Serum--Lactate Dehydrogenase | 0.004400731 |
| 30 | RESULT_40275 | CBC with Plt Count and Auto Diff--Red Cell Distribution Width | 0.004373323 |
| 31 | RESULT_16 | BLOOD GAS ANALYSIS WITH ELECTROLYTES (AEGIS)--PaO2* | 0.004197738 |
| 32 | RESULT_20025 | Basic Metabolic Panel--Creatinine, Serum or Plasma | 0.004190682 |
| 33 | RESULT_29 | BLOOD GAS ANALYSIS WITH ELECTROLYTES (AEGIS)--Glucose | 0.004122638 |
| 34 | RESULT_20002 | Basic Metabolic Panel--Potassium, Serum or Plasma | 0.004028108 |
| 35 | ANESTH_PROV_DWID | ANESTH_PROV_DWID | 0.00401752 |
| 36 | CODE_33954 | ECMO/ECLS INSJ OF PRPH CANNULA 6 YRS&OLDER OPEN | 0.003984761 |
| 37 | RESULT_25 | BLOOD GAS ANALYSIS WITH ELECTROLYTES (AEGIS)--Na+ | 0.00398155 |
| 38 | RESULT_31 | BLOOD GAS ANALYSIS WITH ELECTROLYTES (AEGIS)--BICARBONATE | 0.003938062 |
| 39 | RESULT_20023 | Comprehensive Metabolic Panel--Urea Nitrogen, Serum or Plasma | 0.003839066 |
| 40 | RESULT_20004 | Basic Metabolic Panel--Carbon Dioxide, Serum or Plasma | 0.003792802 |
| 41 | RESULT_40056 | CBC with Plt Count and Auto Diff--Eosinophil # | 0.003786783 |
| 42 | RESULT_36 | BLOOD GAS ANALYSIS WITH ELECTROLYTES (AEGIS)--PaO2 | 0.003776712 |
| 43 | RESULT_40320 | CBC with Platelet Count--White Blood Cell Count | 0.003736109 |
| 44 | CODE_02UA08Z | SUPPLEMENT HEART W/ZOOPLASTIC TISSUE OPEN APPRCH | 0.003732315 |
| 45 | CODE_34716 | OPN AXILLARY/SUBCLAVIAN ART EXPOS W/CNDT CRTJ | 0.003713126 |
| 46 | RESULT_40145 | CBC with Platelet Count--Mean Corpuscular Hemoglobin | 0.00368424 |
| 47 | CODE_33020 | PERICARDIOTOMY REMOVAL CLOT/FOREIGN BODY PRIMARY | 0.003676088 |
| 48 | CODE_33863 | AS-AORT GRF W/CARD BYP & AORTIC ROOT RPLCMT | 0.003607437 |
| 49 | RESULT_20029 | Hepatic Function Panel--Protein Total, Serum/Plasma | 0.003604563 |
| 50 | RESULT_40130 | CBC with Plt Count and Auto Diff--Lymphocyte % | 0.003603129 |
| 51 | CODE_0FY00Z0 | TRANSPLANTATION LIVER ALLOGENEIC OPEN APPROACH | 0.003602635 |
| 52 | CODE_B3101ZZ | FLUOROSCOPY THORACIC AORTA LOW OSMOLAR CONTRAST | 0.003483158 |
| 53 | RESULT_40125 | CBC with Plt Count and Auto Diff--Lymphocyte # | 0.003455465 |
| 54 | CODE_76001 | FLUOROSCOPY SPX >1 HOUR PHYS/QHP TIME | 0.00343085 |
| 55 | CODE_5A1D60Z | PERFORMANCE OF URINARY FILTRATION MULTIPLE | 0.00342089 |
| 56 | RESULT_30220 | PT, International Normalized Ratio--Prothrombin Time | 0.0034177 |
| 57 | RESULT_40170 | CBC with Plt Count and Auto Diff--Monocyte # | 0.00339483 |
| 58 | AGE_AT_ADM | AGE_AT_ADM | 0.003378381 |
| 59 | RESULT_SAMPLENO | BLOOD GAS ANALYSIS WITH ELECTROLYTES (AEGIS)--Unmapped | 0.003365192 |
| 60 | CODE_93321 | DOP ECHOCARD PULSE WAVE W/SPECTRAL F-UP/LMTD STD | 0.003344464 |
| 61 | CODE_99285 | EMERGENCY DEPT VISIT HIGH SEVERITY&THREAT FUNCJ | 0.003340818 |
| 62 | RESULT_20 | BLOOD GAS ANALYSIS WITH ELECTROLYTES (AEGIS)--tHb | 0.003317626 |
| 63 | RESULT_20027 | Basic Metabolic Panel--Calcium, Serum or Plasma | 0.003303256 |
| 64 | RESULT_28 | BLOOD GAS ANALYSIS WITH ELECTROLYTES (AEGIS)--LACTATE | 0.003249643 |
| 65 | CODE_33238 | RMVL PRM TRANSVENOUS ELECTRODE THORACOTOMY | 0.003225195 |
| 66 | CODE_02PA0MZ | REMOVAL OF CARDIAC LEAD FROM HEART OPEN APPROACH | 0.003194527 |
| 67 | RESULT_61204 | Susceptibility - Anaerobe by broth dilution (Billed) , Bacteroides caccae--Moxifloxacin | 0.003179041 |
| 68 | CODE_03QY0ZZ | REPAIR UPPER ARTERY OPEN APPROACH | 0.00317616 |
| 69 | RESULT_40070 | CBC with Plt Count and Auto Diff--Granulocyte # | 0.003084269 |
| 70 | CODE_32482 | RMVL LUNG OTHER THAN PNEUMONECT 2 LOBES BILOBEC | 0.003011849 |
| 71 | CODE_21615 | EXCISION 1ST &/CERVICAL RIB | 0.002993204 |
| 72 | RESULT_70138 | Thyroxine Free--Thyroxine Free | 0.002991766 |
| 73 | CODE_33880 | EVASC RPR DTA COVERAGE ART ORIGIN 1ST ENDOPROSTH | 0.002987436 |
| 74 | RESULT_40032 | CBC with Plt Count and Auto Diff--Basophil % | 0.00297722 |
| 75 | RESULT_50280 | Haptoglobin--Haptoglobin | 0.002943953 |
| 76 | RESULT_20003 | Basic Metabolic Panel--Chloride, Serum or Plasma | 0.0029326 |
| 77 | CODE_85060 | BLOOD SMEAR PERIPHERAL INTERP PHYS W/WRIT REPORT | 0.002920847 |
| 78 | RESULT_2004556 | Fibrinogen Panel--X_FIBAG QL PT | 0.002908609 |
| 79 | RESULT_30223 | PT, International Normalized Ratio--International Normalized Ratio | 0.002908148 |
| 80 | RESULT_30 | BLOOD GAS ANALYSIS WITH ELECTROLYTES (AEGIS)--BE | 0.002901649 |
| 81 | RESULT_70225 | TSH 3rd Generation--TSH 3rd Generation | 0.002891373 |
| 82 | RESULT_2013435 | CBC with Plt Count and Auto Diff--Immature Granulocytes # | 0.0027968 |
| 83 | RESULT_2013369 | CBC with Platelet Count--Nucleated Red Blood Cell # | 0.002782608 |
| 84 | RESULT_32 | BLOOD GAS ANALYSIS WITH ELECTROLYTES (AEGIS)--Hct | 0.002774513 |
| 85 | RESULT_40150 | CBC with Platelet Count--Mean Corpuscular Volume | 0.002735878 |
| 86 | RESULT_20009 | Gamma Glutamyl Transferase, Serum/Plasma--Gamma Glutamyl Transferase | 0.002729666 |
| 87 | CODE_03740DZ | DILAT LT SUBCLAVIAN ARTERY W/INTRALUM DEVC OPEN | 0.002713457 |
| 88 | RESULT_20007 | Comprehensive Metabolic Panel--Aspartate Aminotransferase | 0.002708631 |
| 89 | CODE_95941 | IONM REMOTE/NEARBY/>1 PATIENT IN OR PER HOUR | 0.002677766 |
| 90 | CODE_0232T | NJX PLTLT PLASMA W/IMG HARVEST/PREPARATION | 0.002666985 |
| 91 | CODE_33210 | INSJ/RPLCMT TEMP TRANSVNS 1CHMBR ELTRD/PM CATH | 0.002657881 |
| 92 | RESULT_20314_see note | Urinalysis with Reflex to Culture--UA Blood | 0.002618725 |
| 93 | CODE_03RH0JZ | REPLACEMENT RIGHT CCA W/SYNTH SUBST OPEN APPRCH | 0.002606089 |
| 94 | CODE_50230 | NEPHRECTOMY W/PRTL URETERECT OPEN RIB RESCJ RAD | 0.002585979 |
| 95 | RESULT_40140 | CBC with Plt Count and Auto Diff--Mean Corpuscular HGB Concentration | 0.002585611 |
| 96 | CODE_02UF0KZ | SUPPLEMENT AORTIC VALVE NONAUTO TISS SUBST OPEN | 0.002556969 |
| 97 | RESULT_20005 | Comprehensive Metabolic Panel--Alkaline Phosphatase | 0.002555658 |
| 98 | CODE_99238 | HOSPITAL DISCHARGE DAY MANAGEMENT 30 MIN/< | 0.002544713 |
| 99 | RESULT_20316_negative | Urinalysis, Complete--UA Nitrite | 0.002528873 |
| 100 | RESULT_2013053 | Body Fluid, Lactate Dehydrogenase--Body Fluid, LDH | 0.002525443 |
| 101 | RESULT_20024 | Basic Metabolic Panel--Glucose, Serum or Plasma | 0.002521421 |
| 102 | CODE_4A11X4G | MONITOR PERIPH NERVOUS ELECTRIC ACT INTRAOP EXT | 0.00251771 |
| 103 | RESULT_20008 | Comprehensive Metabolic Panel--Alanine Aminotransferase | 0.002515871 |
| 104 | CODE_0PQ20ZZ | REPAIR 3 OR MORE RIBS OPEN APPROACH | 0.002492732 |
| 105 | CODE_04Q90ZZ | REPAIR RIGHT RENAL ARTERY OPEN APPROACH | 0.002485658 |
| 106 | CODE_5A1221Z | PERFORMANCE OF CARDIAC OUTPUT CONTINUOUS | 0.002471515 |
| 107 | CODE_33988 | INSERT LEFT HEART VENT BY THORACIC INC ECMO/ECLS | 0.0024605 |
| 108 | CODE_04CN0ZZ | EXTIRPATION MATTER LT POPLITEAL ARTERY OPEN | 0.002451236 |
| 109 | RESULT_20037 | Iron and Iron Binding Capacity--Iron, Serum or Plasma | 0.002438589 |
| 110 | CODE_02QC0ZZ | REPAIR LEFT HEART OPEN APPROACH | 0.002429798 |
| 111 | CODE_33975 | INSJ VENTRIC ASSIST DEV XTRCORP SINGLE VENTRICLE | 0.002374355 |
| 112 | CODE_0WCD0ZZ | EXTIRPATION MATTER PERICARDIAL CAVITY OPEN | 0.002373843 |
| 113 | CODE_02VX0DZ | RESTRICTION THOR AORTA ASCEND/ARCH IL DEVC OPEN | 0.002342296 |
| 114 | RESULT_70428 | Hemoglobin A1C--Hemoglobin A1C | 0.002335709 |
| 115 | RESULT_70145 | Thyroid Stimulating Hormone--Thyroid Stimulating Hormone | 0.002323091 |
| 116 | CODE_27602 | DCMPRN FASCT LEG ANT&/LAT&PST CMPRT | 0.00230836 |
| 117 | CODE_95938 | SHORT-LATENCY SOMATOSENS EP STD UPR & LOW LIMB | 0.002301853 |
| 118 | CODE_35226 | RPR BLOOD VESSEL DIRECT LOWER EXTREMITY | 0.002263373 |
| 119 | CODE_32.50 | THORACOSCOPIC PNEUMONECTOMY | 0.002252435 |
| 120 | RESULT_19 | BLOOD GAS ANALYSIS WITH ELECTROLYTES (AEGIS)--METHb | 0.002199716 |
| 121 | RESULT_2013361 | CBC with Platelet Count--Immature Platelet Fraction | 0.00219706 |
| 122 | RESULT_20304 | Urinalysis, Complete--UA Specific Gravity | 0.00219238 |
| 123 | RESULT_20032 | Comprehensive Metabolic Panel--Bilirubin, Total, Serum or Plasma | 0.0021814 |
| 124 | CODE_02HK0DZ | INSERTION INTRALUMINAL DEVICE RT VENTRICLE OPEN | 0.002175591 |
| 125 | CODE_3E0F8GC | INTRO OTH TX SBSTNC RESP TRACT NAT OPENING ENDO | 0.002163247 |
| 126 | RESULT_27 | BLOOD GAS ANALYSIS WITH ELECTROLYTES (AEGIS)--Ca++ | 0.002160495 |
| 127 | RESULT_30235 | Partial Thromboplastin Time--Partial Thromboplastin Time | 0.002149544 |
| 128 | RESULT_21 | BLOOD GAS ANALYSIS WITH ELECTROLYTES (AEGIS)--O2 CONTENT | 0.002121239 |
| 129 | RESULT_20058 | Hemoglobin, Plasma--Hemoglobin, Plasma | 0.00211269 |
| 130 | RESULT_13 | BLOOD GAS ANALYSIS WITH ELECTROLYTES (AEGIS)--FI02 | 0.002106007 |
| 131 | RESULT_18 | BLOOD GAS ANALYSIS WITH ELECTROLYTES (AEGIS)--COHb | 0.002082818 |
| 132 | CODE_84484 | ASSAY OF TROPONIN QUANTITATIVE | 0.002078011 |
| 133 | RESULT_70130 | Testosterone Free and Total, Adult Male--Testosterone, Adult Male | 0.002077804 |
| 134 | RESULT_23 | BLOOD GAS ANALYSIS WITH ELECTROLYTES (AEGIS)--A-aO2 | 0.002074792 |
| 135 | SURGEON_PROV_DWID | SURGEON_PROV_DWID | 0.002061953 |
| 136 | CODE_99356 | PROLONGED SERVICE I/P REQ UNIT/FLOOR TIME 1ST HR | 0.002044062 |
| 137 | CODE_40.29 | SIMPLE EXCISION OF OTHER LYMPHATIC STRUCTURE | 0.00202142 |
| 138 | RESULT_40175 | CBC with Plt Count and Auto Diff--Monocyte % | 0.002020885 |
| 139 | CODE_85576 | PLATELET AGGREGATION IN VITRO EACH AGENT | 0.002019303 |
| 140 | RESULT_99085_orange | Cell Count, Body Fluid--Color | 0.002018484 |
| 141 | CODE_85520 | HEPARIN ASSAY | 0.002017446 |
| 142 | CODE_81002 | URNLS DIP STICK/TABLET RGNT NON-AUTO W/O MICRSCP | 0.001989079 |
| 143 | RESULT_20090 | Hepatitis B Virus Surface Antibody--Hepatitis B Surface Antibody | 0.001976625 |
| 144 | RESULT_2000075 | FFP REQ--Unit Number | 0.00197547 |
| 145 | RESULT_20312_negative | Urinalysis with Reflex to Culture--UA Bilirubin | 0.001973177 |
| 146 | CODE_0BBM0ZZ | EXCISION OF BILATERAL LUNGS OPEN APPROACH | 0.001963774 |
| 147 | CODE_0TT00ZZ | RESECTION OF RIGHT KIDNEY OPEN APPROACH | 0.001951056 |
| 148 | CODE_0FQ10ZZ | REPAIR RIGHT LOBE LIVER OPEN APPROACH | 0.001936015 |
| 149 | CODE_04CK0ZZ | EXTIRPATION MATTER RT FEMORAL ARTERY OPEN APPRCH | 0.001935988 |
| 150 | CODE_02UG08Z | SUPPLEMENT MITRAL VALVE ZOOPLASTIC TISSUE OPEN | 0.001921039 |
| 151 | RESULT_20028 | Phosphorus, Inorganic, Plasma or Serum--Phosphorus, Inorganic, Serum or Plasma | 0.001910164 |
| 152 | RESULT_20001 | Basic Metabolic Panel--Sodium, Serum or Plasma | 0.001904206 |
| 153 | CODE_85390 | FIBRINOLYSINS/COAGULOPATHY SCREEN INTERP&REPOR | 0.001895995 |
| 154 | CODE_0W9D3ZZ | DRAINAGE PERICARDIAL CAVITY PERQ | 0.00187874 |
| 155 | RESULT_20315_negative | Urinalysis with Reflex to Culture--UA Leukocyte Esterase | 0.001873919 |
| 156 | RESULT_20031 | Lipid Panel--Cholesterol, Serum or Plasma | 0.001864996 |
| 157 | RESULT_30135 | Fibrinogen Panel--Fibrinogen Ag | 0.001844277 |
| 158 | SURGEON_PROV_DWID_279156 | SURGEON_PROV_DWID_279156 | 0.001842652 |
| 159 | CODE_06HY33Z | INSERTION INFUSION DEVICE INTO LOWER VEIN PERQ | 0.001826604 |
| 160 | RESULT_20033 | Hepatic Function Panel--Bilirubin Direct | 0.001824747 |
| 161 | CODE_02PA0RZ | REMOVAL SHORT-T EXT HEART ASSIST SYS HEART OPEN | 0.001823792 |
| 162 | CODE_33944 | BKBENCH PREPJ CADAVER DONOR HEART ALLOGRAFT | 0.001819246 |
| 163 | RESULT_2099892 | Hepatitis A Virus Antibody, IgM--NC_HAVM Result 1 | 0.001813802 |
| 164 | CODE_0GB30ZZ | EXCISION OF RIGHT ADRENAL GLAND OPEN | 0.001808836 |
| 165 | CODE_0BBN0ZZ | EXCISION OF RIGHT PLEURA OPEN APPROACH | 0.001806887 |
| 166 | CODE_35661 | BYP OTH/THN VEIN FEMORAL-FEMORAL | 0.00179504 |
| 167 | CODE_02L70DK | OCCLUSION LT ATRIAL APPENDAGE INTRALUM DEVC OPEN | 0.001786002 |
| 168 | RESULT_2006109 | Thyroid Stim. Hormone w/Rflx to Free T4--Thyroid Stim. Hormone w/Rflx to Free T4 | 0.001753865 |
| 169 | CODE_39.61 | EXTRACORPOREAL CIRC AUXILIARY OPEN HEART SURGERY | 0.001742748 |
| 170 | RESULT_2004567 | Fibrinogen Panel--X_FIB QL PT | 0.001729781 |
| 171 | RESULT_99597 | Measles (Rubeola) Abs, IgG and IgM--Measles, Rubeola, Antibody IgM | 0.001723561 |
| 172 | RESULT_70427 | Hemoglobin A1C--Estimated Average Glucose | 0.001718207 |
| 173 | RESULT_2004555 | Thrombosis, Uncommon Etiologies--X_PR S FR QL PT | 0.00171158 |
| 174 | CODE_88366 | IN SITU HYBRIDIZATION EA MULTIPLEX PROBE STAIN | 0.00170494 |
| 175 | CODE_0BH17EZ | INSERT ENDOTRACHL AIRWAY TRACHEA NAT/ART OPENING | 0.001693641 |
| 176 | CODE_35271 | RPR BLOOD VSL GRF OTH/THN VEIN INTRATHRC W/BYP | 0.001688587 |
| 177 | CODE_04CH3ZZ | EXTIRPATION MATTER RT EXT ILIAC ART PERQ APPRCH | 0.001673833 |
| 178 | RESULT_2013370 | CBC with Platelet Count--Nucleated Red Blood Cells % | 0.001669856 |
| 179 | CODE_33530 | ROPRTJ CAB/VALVE PX > 1 MO AFTER ORIGINAL OPERJ | 0.001663809 |
| 180 | RESULT_10487_billed | RBCLR Product--BILL_RBC L-R | 0.00163903 |
| 181 | RESULT_2002404 | Hepatitis Panel, Acute--Hepatitis C Antibody by CIA Index | 0.001619765 |
| 182 | CODE_0Y970ZZ | DRAINAGE RIGHT FEMORAL REGION OPEN | 0.001616546 |
| 183 | RESULT_20302_green | Urinalysis with Reflex to Culture--UA Color | 0.001596877 |
| 184 | CODE_93312 | ECHO TRANSESOPHAG R-T 2D W/PRB IMG ACQUISJ I&R | 0.001592266 |
| 185 | RESULT_90613 | Troponin I--Troponin-I | 0.001590759 |
| 186 | CODE_031H0AJ | BP RT CCA RT EXTRACRANIAL ART AUTO ART TISS OPEN | 0.00159062 |
| 187 | RESULT_2013985_negative | Extended Myositis Panel--X_SSc NOR90 | 0.00158681 |
| 188 | RESULT_95011 | Cell Count, Body Fluid--% Eosinophils fluid | 0.001572122 |
| 189 | RESULT_20045 | Lactic Acid, Plasma--Lactic Acid, Plasma | 0.001568911 |
| 190 | CODE_33237 | RMVL PRM EPICAR PM&ELTRDS THORCOM DUAL LEAD SY | 0.001567327 |
| 191 | RESULT_30130 | Fibrinogen--Fibrinogen | 0.001562933 |
| 192 | RESULT_40185 | Manual Differential Confirmation--Myelocyte Manual | 0.001550877 |
| 193 | RESULT_50390 | Mumps Virus Ab, IgG--Mumps Virus Antibody, IgG | 0.001529102 |
| 194 | RESULT_BE | RBC REQ--EXPDATE | 0.001507563 |
| 195 | CODE_4A133B1 | MONITORING ARTERIAL PRESSURE PERIPHERAL PERQ | 0.001503819 |
| 196 | CODE_041L0JK | BYPASS LT FEMORAL ART BIL FEM ART SYNTH SUBS OPN | 0.001503717 |
| 197 | CODE_06BS0ZZ | EXCISION LEFT LESSER SAPHENOUS VEIN OPEN APPROCH | 0.001500734 |
| 198 | RESULT_97181_rescheduled | Turnback--Turnback Reason Prompt | 0.001495741 |
| 199 | CODE_47135 | LVR ALTRNSPLJ ORTHOTOPIC PRTL/WHL DON ANY AGE | 0.001474001 |
| 200 | RESULT_50065 | Mitochondrial M2 Ab, IgG--Mitochondrial (M2) Antibody, IgG | 0.001470637 |
| 201 | GENDER_CODE | GENDER_CODE | 0.001467467 |
| 202 | RESULT_2003395 | Cytology, Non-Gynecologic | 0.001464128 |
